# Role of c-Jun N-Terminal Kinases (JNKs) in Epilepsy and Metabolic Cognitive Impairment

**DOI:** 10.3390/ijms21010255

**Published:** 2019-12-30

**Authors:** Oriol Busquets, Miren Ettcheto, Amanda Cano, Patricia R. Manzine, Elena Sánchez-Lopez, Triana Espinosa-Jiménez, Ester Verdaguer, Rubén Dario Castro-Torres, Carlos Beas-Zarate, Francesc X. Sureda, Jordi Olloquequi, Carme Auladell, Jaume Folch, Antoni Camins

**Affiliations:** 1 Department of Pharmacology, Toxicology and Therapeutic Chemistry, Faculty of Pharmacy and Food Science, University of Barcelona, 08028 Barcelona, Spain; oriolbusquets92@gmail.com (O.B.); e_miren60@hotmail.com (M.E.); triana.espinosa95@hotmail.com (T.E.-J.); rubendario230@gmail.com (R.D.C.-T.); 2 Department of Biochemistry and Biotechnology, Faculty of Medicine and Life Science, University Rovira i Virgili, 43201 Reus, Spain; francesc.sureda@urv.cat (F.X.S.); jaume.folch@urv.cat (J.F.); 3 Biomedical Research Networking Centre in Neurodegenerative Diseases (CIBERNED), Instituto de Salud Carlos III, 28029 Madrid, Spain; amanda90cf@gmail.com (A.C.); everdaguer@ub.edu (E.V.); cauladell@ub.edu (C.A.); 4 Institute of Neuroscience (Neuro UB), University of Barcelona, 08028 Barcelona, Spain; 5 Institute of Nanoscience and Nanotechnology (IN2UB), University of Barcelona, 08028 Barcelona, Spain; esanchezlopez@ub.edu; 6 Department of Pharmacy, Pharmaceutical Technology and Physical Chemistry, Faculty of Pharmacy and Food Science, University of Barcelona, 08028 Barcelona, Spain; 7 Department of Gerontology, Federal University of São Carlos (UFSCar), São Carlos 13565-905, Brazil; pmanzine@ufscar.br; 8 Department of Cellular Biology, Physiology and Immunology, Faculty of Biology, University of Barcelona, 08028 Barcelona, Spain; 9 Department of Cellular and Molecular Biology, C.U.C.B.A., University of Guadalajara and Neuroscience Division, Sierra Mojada, Col. Independencia, Zapopan, Guadalajara, Jalisco 45200, Mexico; carlosbeas55@gmail.com; 10 Laboratory of Cellular and Molecular Pathology, Instituto de Ciencias Biomédicas, Facultad de Ciencias de la Salud, Universidad Autónoma de Chile, Talca 3460000, Chile; jordiog82@gmail.com

**Keywords:** brain, epilepsy, metabolism, type 2 diabetes, c-Jun-N-terminal kinase, JNK inhibitor, cognitive impairment

## Abstract

Previous studies have reported that the regulatory function of the different c-Jun N-terminal kinases isoforms (JNK1, JNK2, and JNK3) play an essential role in neurological disorders, such as epilepsy and metabolic-cognitive alterations. Accordingly, JNKs have emerged as suitable therapeutic strategies. In fact, it has been demonstrated that some unspecific JNK inhibitors exert antidiabetic and neuroprotective effects, albeit they usually show high toxicity or lack therapeutic value. In this sense, natural specific JNK inhibitors, such as Licochalcone A, are promising candidates. Nonetheless, research on the understanding of the role of each of the JNKs remains mandatory in order to progress on the identification of new selective JNK isoform inhibitors. In the present review, a summary on the current gathered data on the role of JNKs in pathology is presented, as well as a discussion on their potential role in pathologies like epilepsy and metabolic-cognitive injury. Moreover, data on the effects of synthetic small molecule inhibitors that modulate JNK-dependent pathways in the brain and peripheral tissues is reviewed.

## 1. Introduction

Modern societies are characterized by technological innovations that have transformed human life in fundamental ways. In developed countries, people live longer and are healthier. Now, it is possible to cure diseases that were once considered death sentences. Still, there are significant woes that need to be addressed.

An increase in stress and anxiety, unbalanced and unhealthy diets, sedentary lifestyles and exposure to pollution and radiation are just a few of these struggles, and they have paramount impact on human health. Understanding how these factors influence the development of complex diseases is crucial in order to develop safe, state-of-the-art treatments. As that the molecular bases of human diseases start to be uncovered, a better understanding of these phenomena is emerging, and the development of promising treatment strategies.

In this regard, some of the paramount signaling pathways involved in cell regulation are behind the most prevalent pathologies. The mitogen-activated protein kinases (MAPKs) are a superfamily of major signaling pathways responsible for the regulation of several physiological functions. However, when dysregulated, they become significant physiological dysregulators and, therefore, potential therapeutic targets.

## 2. Mitogen Activated Protein Kinases (MAPKs)

The maintenance of homeostasis is a key aspect of cellular physiology. This is possible thanks to some highly conserved biochemical mechanisms that respond to intra- and extracellular stimuli through regulatory modifications.

The MAPKs are a superfamily of intracellular highly conserved serine (Ser)/threonine (Thr) kinases that regulate multiple cellular functions by converting a diverse plethora of hormonal, chemical, and physical stimuli into cellular responses. They are located in most eukaryotic cells and are responsible for the control of cell proliferation, differentiation, migration, senescence, and apoptosis, among other mechanisms [1]. The substrates for these kinases are present in the cytoplasm, mitochondria, Golgi apparatus, endoplasmic reticulum (ER), and the nucleus. The signals transmitted via the different cascades modulate the activity of many transcription factors, transcription suppressors, and chromatin remodeling proteins [2].

**Figure 1:**
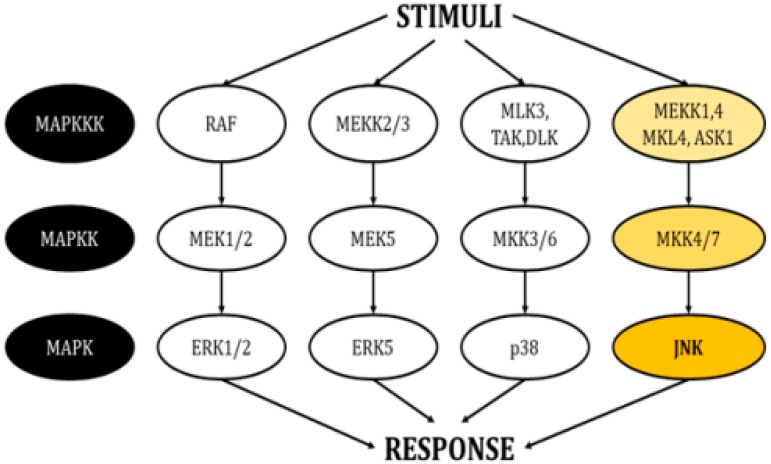
Schematic of the cascades in the MAPK pathway. Each pathway responds to different stimuli and creates an appropriate responses (modified from Cargnello, M. and Roux, P.P., 2011) [1].

This family of kinases forms four main cascades: The signal-related kinases (ERK) 1/2, ERK5, p38, and the c-Jun N-terminal kinases (JNKs) (Figure 1) [3,4]. All these cascades propagate signals through sequential phosphorylation aided by scaffold proteins and have the ultimate role of inducing modifications in gene expression. They also interact with proteins in the plasma membrane and the cytosol, thereby regulating additional cellular mechanisms.

Each MAPK cascade responds to different stimuli, allowing the generation of appropriate responses according to the environment surrounding the cell. In general, the activity of ERK1/2 is controlled by growth factors, insulin, ligands linked to the activity of G-protein-coupled receptors, cytokines, osmotic pressure, and microtubule disorganization [5]. In turn, growth factors, oxidative stress, and hyperosmolarity are able to stimulate ERK5 [6]. The p38 isoforms respond severely to environmental agents like oxidative stress and UV radiation, as well as to inflammatory cytokines [7]. Finally, JNKs, also known as stress-activated protein kinases (SAPKs), are triggered by multiple stimuli, including heat shock, ionizing radiation, oxidative stress, deoxyribonucleic acid (DNA)-damaging agents, cytokines, ultraviolet irradiation, protein inhibitors, G-protein-coupled receptors, deprivation, and the presence of growth factors [8,9].

The present review focuses on JNKs, due to their ability to control a wide range of cellular mechanisms in physiological and pathological conditions, as well as for their potential as therapeutic targets.

## 3. C-JUN N-Terminal Kinases (JNKs)

The JNKs are stress-response elements that become active through dual phosphorylation in threonine (Thr) and tyrosine (Tyr) residues by the mitogen-activated protein kinase (MKK) 4 and 7 with the JNK-interacting scaffold protein (JIP) [10]. The JNK family accounts for three isoforms (JNK1, JNK2, and JNK3), which share an 85% identity and are codified by three different genes, Mapk8 (*Jnk1*), Mapk9 (*Jnk2*), and Mapk10 (*Jnk3*), which can generate up to 10 spliced protein products with molecular masses ranging from 46 to 55 KDa. Isoforms JNK1 and JNK2 are present almost ubiquitously in all tissues while only the brain, along with testis and cardiac myocytes, expresses JNK3 [9,11]. During embryonic development in murine models, isoforms JNK1 and JNK2 are the first expressed at E7 while JNK3 starts its expression at E11 [12,13]. Studies using knockout (KO) mice have determined that the deficiency of any of the three isoforms is viable, without apparent morphological abnormalities in early developmental stages. On the contrary, the lack of expression of both Mapk8 (JNK1) and Mapk9 (JNK2) is non-viable. In turn, in vitro triple JNK KO showed neuronal hypertrophy and a reduction in the number of dendrites but also an increased lifespan due to a reduction of the capacity to activate apoptosis [12]. This indicates that isoforms can compensate each other’s functions during development and that one of them probably has a more significant role than the others.

In vitro studies have shown that JNK activation can be transient, often leading to cellular survival signals, or sustained, leading to cell death. In most cases, when activated, these kinases re-localize from the cytoplasm into the nucleus, where they phosphorylate the c-JUN transcription factor in the Ser63/73 residues [3]. Additional substrates for the JNKs include p53, activating transcription factor 2, heat shock factor 1, c-Myc and JUNB, among others. Continuous discoveries, the description of new mechanisms, and the widespread effects of these kinases suggest that there are still many other substrates to be identified [1,9].

### 3.1. Role of JNKs in Subcellular Compartments

**Figure 2:**
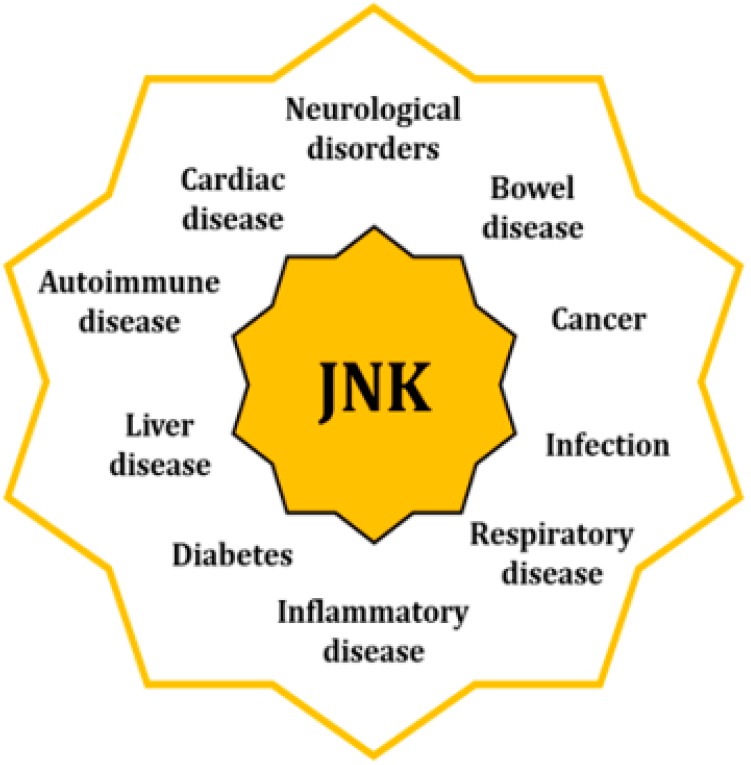
Increased activity of the JNKs is associated with pathologies of different nature through their regulatory control of multiple cell mechnisms.

The fact that JNK activity is upregulated in several disorders underpins their significant role as originators and aggravators of pathological features [3] (Figure 2). When cells are exposed to a physical or chemical stress, the initial response activates survival mechanisms; however, prolonged stress eventually leads to cellular death [14]. Specifically, sustained activation of the JNKs favors the development of apoptotic and necrotic cellular mechanisms, coupled with an upregulated expression of cytotoxic ligands like tumor necrosis factor (TNF)-α or interleukin (IL)-1β, as well as an increased concentration of reactive oxygen species (ROS) [15,16,17]. In addition, JNK activities regulate the intrinsic apoptotic pathway through mitochondria by modulating the activity of the Bcl2 family members, hence activating pro-apoptotic agents and inhibiting the anti-apoptotic ones [18].

Pathologies linked to the activity of the JNKs can be found in almost all systems and apparatus, such as neurological, coronary, hepatobiliary, and respiratory. JNKs are also involved in autoimmune and inflammatory disorders, as well as in cancer, diabetes, and obesity. In many cases, they are associated with dysregulations of mitochondria and ER, as well as with the alteration of other cellular mechanisms like autophagy or cell cycle progression [3,9].

#### 3.1.1. JNK Activation Alters Mitochondrial Activity

Mitochondria are double membrane-bounded organelles with a critical role in the regulation of many cellular processes [19]. Their energetic function depends upon the production of molecular intermediaries in the Krebs’s cycle and the maintenance of chemiosmotic balances between the inner and intermembrane spaces, which allows the synthesis of adenosine triphosphate (ATP) through oxidative phosphorylation (OXPHOS) [20].

**Figure 3:**
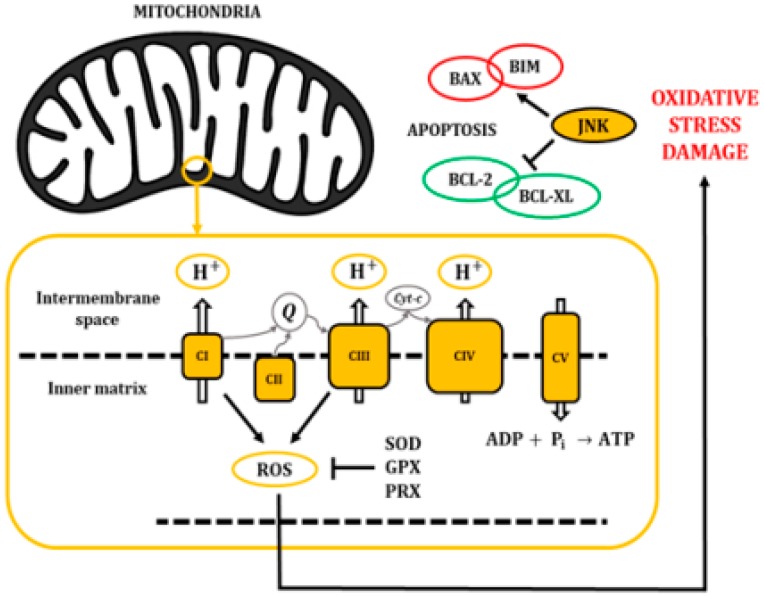
The electron transport chain pumps H^+^ from the cytosol into the intermembrane space of the mitochondria, creating a gradient that returns into the inner matrix through the ATP synthase (CV) and allowing the synthesis of ATP. Accumulation of ROS leads to oxidative stress and damage to macromolecules, inducing pro-apoptotic activity through the activation of JNKs.

The mitochondrial electron transport chain efficiently pumps protons (H^+^) into the intermembrane space; however, some electrons leak into the inner matrix, becoming a source for the generation of reactive oxygen species (ROS) (Figure 3). In fact, there is growing evidence indicating that most of the superoxide radicals are generated by the OXPHOS complexes I and III [21]. ROS are usually defined as partially reduced metabolites of oxygen with a higher reactivity than its precursors [22]. It has been shown that about 1% to 4% of the O_2_ that is used in cellular respiration is converted into O_2_^−^, which may be later transformed into hydrogen peroxide, hydroxyl radicals, or peroxynitrite [23,24].

In physiological conditions, the concentration of ROS follows a tightly controlled pattern of transient fluctuations with regulatory functions [25]. Organisms have evolved multiple mechanisms to counteract the production and accumulation of ROS. These pathways include non-enzymatic molecules like glutathione, vitamins A, C, and E and, flavonoids, as well as enzymatic ROS-degrading agents like the superoxide dismutase (SOD), catalase, glutathione peroxidase (GPX), and peroxiredoxin (PRX). Additional antioxidant protection can be achieved through the activity of L-γ-glutamyl-L-cysteinylglycine (GSH)-linked antioxidant enzymes [26].

Unfortunately, the mechanisms responsible for the degradation of ROS are not always enough to counteract their production, causing an increase in their concentration that may cause severe damage to most macromolecules. This situation is known as oxidative stress [25]. Hence, ROS can damage DNA by direct oxidation, and inactivate iron-sulphur (Fe-S) proteins. Moreover, they can cause lipid peroxidation, which can affect virtually all membrane-dependent functions [26].

Importantly, oxidant injury elicits a wide spectrum of responses, ranging from growth arrest to cell death, through the release of pro-apoptotic proteins from the intermembrane space of mitochondria. It has been described that oxidative stress activates JNKs and would play a major role in this mitochondrial mechanism of apoptosis regulation [25,26]. Specifically, the JNK cascade phosphorylates pro-apoptotic proteins like the B-cell lymphoma 2-like protein 4 (BAX) or B-cell lymphoma 2-like protein 11 (BIM). In addition, it can phosphorylate the anti-apoptotic protein B-cell lymphoma 2 (BCL-2) and B-cell lymphoma extra-large (BCL-XL) to induce their inhibition. Eventually, these mechanisms affect the mitochondrial membrane integrity and cause the release of cytochrome c, second mitochondria-derived activator of caspases (SMACs), and direct IAP-binding protein with Low PI (DIABLO) proteins (Figure 3) [27,28].

#### 3.1.2. Endoplasmic Reticulum Stress (ERS) Activates JNK

**Figure 4:**
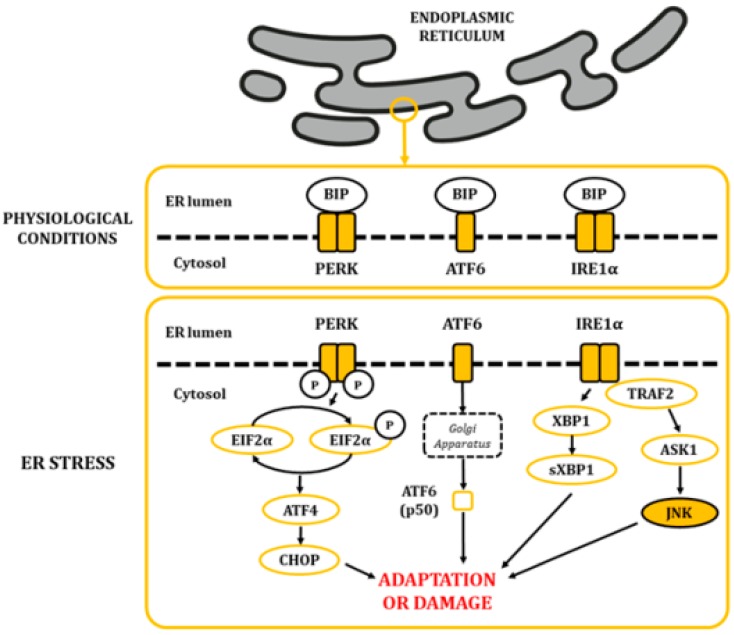
The alteration in ER homeostasis favours the accumulation of unfolded and misfolded proteins in the lumen of the ER. This situation leads to the dissociation of BIP from the PERK, ATF6 and IRE1α proteins, allowing their activation and posterior signaling of the UPR.

The ER is a highly dynamic organelle found in all eukaryotic cells. It is made up of membranous structures that are interconnected and branched to form tubules, vesicles, and cisternae [29]. Its lumen provides a subcellular oxidative compartment that allows the formation of disulphide bonds and proper protein folding [29,30]. Also, it participates in other major cell functions, such as the synthesis of fatty acids and phospholipids, assembly of lipid bilayers, carbohydrate metabolism, and calcium homeostasis [29]. Since proper ER functionality depends on the maintenance of homeostatic conditions, it can be significantly affected by a diverse range of factors, like oxygen pressure, temperature, acidosis, glucose and calcium levels, and energy balance, among others [29]. The lumen of the ER is rich in calcium-dependent chaperones, such as the master regulator immunoglobulin heavy chain-binding protein (BIP; also known as glucose-regulated protein 78 (GRP78) [31]. In physiological conditions, some BIP molecules are bound to specific transmembrane proteins in their N-terminal region, thus maintaining them in an inactive state. These proteins are the protein kinase activated by double-stranded RNA (PKR)-like ER kinase (PERK), the inositol-requiring kinase/endoribonuclease 1 (IRE1), and activating transcription factor 6 (ATF6) [30]. When ER balances are disturbed, protein folding is negatively affected, leading to an accumulation of unfolded and misfolded proteins. This favors the release of BIP, triggering the unfolded protein response (UPR) through the release and activation of PERK, IRE1, and ATF6 (Figure 4) [32,33,34].

Upon PERK pathway activation, PERK subunits homodimerize and autophosphorylate. Then, they phosphorylate the eukaryotic initiation factor 2 alpha (EIF2α) in serine residues, causing a decrease in global protein synthesis. This step is expected to reduce protein influx into the ER, thus ameliorating cytotoxic damage caused by accumulated proteins [35]. Furthermore, it increases the translation of the activating transcription factor 4 (ATF4), which translocates into the nucleus and stimulates genes related to the recovery of homeostasis and/or the adaptation to the environment [36]. Regarding ATF6, once released, it translocates to the Golgi apparatus, where it becomes proteolytically cleaved and releases a p50 fragment from ATF6. P50 then migrates into the nucleus, where it stimulates the expression of genes linked to the stress response [37,38,39]. Finally, homodimerization and autophosphorylation of IRE1α leads to the cleavage of X-box-binding protein 1 (XBP1) mRNA molecules. The spliced products are powerful transcription factors that control the expression of genes that stimulate ER-associated protein degradation [40]. Moreover, IRE1α also activates a cascade through TNF receptor-associated factor 2 (TRAF2) that results in the activation of the JNKs [41].

When stresses are mild or long-lasting, activation of this mechanism allows the adaptation or neutralization of the stress. By contrast, under severe or prolonged stress, UPR favors the activation of pro-apoptotic molecules, leading to the death of dysfunctional cells [29,30]. For example, the C/EBP homologous protein (CHOP) is a key initiator of pro-apoptotic mechanisms, which becomes highly expressed by the activation of the PERK pathway through ATF4. CHOP stimulates the expression of BIM, BID, and BAX and promotes the repression of anti-apoptotic BCL-2. This mechanism is highly coordinated in parallel to the activity of JNKs [42]. In addition, signaling of the IRE1α pathway promotes cell death by activating caspases, a large family of cysteine proteases that either relay or act as the final effectors of apoptosis [38].

Additional consequences of JNK activation may cause other alterations. As an example, when ER stress leads to JNKs’ activation through the IRE1α pathway, BCL-2 activity is suppressed, disrupting its binding to beclin 1, an essential autophagy regulator [43].

### 3.2. JNKs in the Brain

It has been described that JNKs have higher activity in the brain than in any other tissue, which suggests they are key components of protein function and gene expression regulation in the central nervous system [12,44,45]. Specifically, each of the three JNK genes show the highest expression in the neocortex, followed by the hippocampus, thalamus, and midbrain [11].

Studies based on in situ hybridization in mice determined that the JNK isoforms are differently distributed in the adult brain tissue. Hence, JNK1 is found in the somatic space, axons, and dendrites of the cortical layers III and IV. Also, it is significantly highly expressed in the cytosol and dendrites of thalamic neurons. In turn, JNK2 is found in the same areas, but its localization is limited to the cytosol and nucleus. Regarding JNK3, it is found at the highest concentration in layers III and V (pyramidal neuron layers) in the cortex and in the nuclei of Purkinje cells. Finally, isoforms 1 and 3 are the most predominant in the hippocampus, with JNK1 expression being higher in the cornu ammonis 3 (CA3), CA4, and hilus of the dentate gyrus (DG) while JNK3 is mostly localized to the somata of hippocampal pyramidal neurons [46]. On a cellular level, JNKs are primarily distributed in the cytoplasm, but, as it has been previously mentioned, they can also be found in other cellular compartments. Specifically, in neurons, JNKs are located in peripheral structures like dendritic spines and synapses, underpinning their relevance in the maintenance and dynamics of these structures [47].

JNKs play roles in development, cellular proliferation, differentiation, and morphogenesis, as well as in neuronal pathfinding and the maintenance of synapses. In addition, it is thought that they regulate neuronal migration by phosphorylating cytoskeletal proteins. Furthermore, they are involved in neuronal death through apoptotic mechanisms after cellular insults, suggesting that they play important roles in neurodegenerative pathologies and in neuropsychiatric disorders [9,11,48].

JNKs are also involved in the development of inflammatory responses, a feature that is accepted to play an important role in neurodegeneration. Hence, JNKs are expressed in microglia, astrocytes, and oligodendrocytes, which are cells that contribute to this mechanism [49,50]. In the initial stages, the inflammatory response activates microglial cells, leading to the release of cytokines that activate different cells through the upregulation of factors like cJUN and nuclear factor kappa-light-chain-enhancer of activated B cells (NFκB) [12].

## 4. Role of the JNKs in the Development of Disease

In the present work, the relevance of the JNKs in the neuropathophysiology of two different diseases is reviewed: Temporal lobe epilepsy (TLE) and metabolic-related late-onset non-familiar sporadic dementia.

### 4.1. Temporal Lobe Epilepsy

Epilepsy is a chronic, neurological disease that affects people of all ages and is characterized by a predisposition for the occurrence of recurrent seizures [51,52,53]. Epidemiological data indicates that it contributes to 1% to 2% of the global burden of disease. In 2015, the CDC detected that 1.2% of the US had active epilepsy (about 3 million adults and 470,000 children [52,53,54]. A seizure is a transient occurrence of a clinical manifestation of an abnormal, excessively synchronous neuronal activity in the brain. Seizures may be focal when they originate within networks restricted to one cerebral hemisphere or generalized when they rapidly engage networks extended over both cerebral hemispheres [5].

Temporal lobe epilepsy is the most common type of epilepsy in adults and is characterized by an affectation of the temporal lobe, especially the hippocampus and amygdala. It has been described that up to 30% of patients with TLE can develop a refractory epilepsy process [55]. In addition, the existence of uncontrolled seizures ultimately leads to characteristic structural changes, such as hippocampal sclerosis. However, the molecular mechanisms leading to TLE are not fully elucidated.

Neuroanatomical examinations of patients with TLE have shown that they typically display sclerosis in the CA1, CA3, and hilus of the DG of the hippocampus, coupled with granule cell dispersion and sprouting of aberrant mossy fibers in the molecular layer of the DG [56]. TLE also causes cognitive sequelae on memory, attention, language, praxis, executive function, judgment, insight, and problem solving [57,58].

Current standardized treatments for epilepsy include a wide group of molecules with multiple mechanisms of action (antiepileptic drugs; AEDs), but, in about 30% of patients affected by this illness, the pathology becomes pharmacoresistant or refractory. It has been stipulated that this outcome is the consequence of a lack of understanding of all the mechanisms involved in the pathology, as well as due to the nature of AEDs that treat only the symptoms and not the underlying pathological mechanisms. In this situation, surgical resection of the affected tissue is the only alternative, but, in some occasions, it is impracticable or it only reduces the occurrence of seizures [52,56,59,60,61].

On a molecular level, TLE is characterized by profound alterations in cellular homeostasis. As it has previously been mentioned, one of the consequences of seizure activity is the development of sclerotic tissue, derived from cellular death in neural tissue, coupled with inflammatory and immunological reactions [62,63]. The appearance of gliosis, characterized by the proliferation and hypertrophy of glial cells, increases tissue damage and promotes the secretion of cytokines and chemokines (IL1, TNFα, IL6, IL10, interferon α and β, etc.). Crosstalk between neurons and glial cells in this situation reduces the seizure threshold in neurons [64]. Moreover, seizures trigger the apoptotic extrinsic pathway, through the activation of response elements like the TNF receptor 1. Furthermore, the intrinsic pathway is activated as a result of high levels of calcium in the cytoplasmic compartment. This accumulation has negative consequences on a diverse array of organelles, including the mitochondria and ER, resulting in increased production and release of molecules like ROS and cytochrome c, and the upregulation of caspase activity.

The activation of IRE1α in the ER, as well as the release of ROS in the mitochondria, also favors the maintenance of a constant activated state of the JNKs [19,64,65,66]. Indeed, Liu and colleagues [62] demonstrated a significant increase in the IRE1-JNK response in the hippocampi of human patients suffering from TLE. In a rodent study evaluating miRNA expression patterns among different brain regions affected in TLE epileptogenesis, Gorter and colleagues found increased hippocampal miRNAs involved in the MAPK pathway and inflammation [67]. They suggested that these miRNAs could be adequate target genes that would allow the development of a promising therapeutic strategy to prevent epileptogenesis. Moreover, they also evaluated the expression in the blood of miRNAs that could be useful as suitable potential biomarkers of the disease.

Activation of JNK was also observed in experimental models of temporal lobe seizures. Tai and colleagues found a significant JNK hyperactivation in rats treated with intraperitoneal administration of pilocarpine hydrochloride (385 mg/kg) [66]. In another series of experiments, rats were treated with anisomycin, a nonspecific MAPK activator, which caused an increase in seizure frequency. To confirm the hypothesis that JNK overactivation could be relevant in chronic epilepsy, a wide-spectrum unspecific JNK inhibitor (SP600125) was administered and the same authors reported a significant reduction of the convulsive seizure frequency [66]. Regarding this, it also has been reported that anisomycin mediates JNK activation through overexpression of JNK-interacting protein 3 (JIP3), a scaffold protein that is involved in the regulation of the apoptotic process [68]. Interestingly, Wang and colleagues reported a selective neuronal localization of JIP3 in TLE patients and in rodents after post-seizure [69]. Likewise, selective inhibition of JIP3 through the administration of a lentivirus (LV-375JIP3-RNAi) decreased seizure severity mediated by experimental convulsiveness [69].

The use of preclinical models of TLE has allowed the roles of the different JNK isoforms in this disease to be described. Specifically, combining TLE preclinical models with isoform-specific JNK KOs has revealed that the absence of JNK1 and JNK3 renders neuroprotection in mice [11,70,71,72,73,74]. Furthermore, it has been demonstrated that the absence of these isoforms also reduces the occurrence and severity of seizures, glial reactivity, and the expression of proinflammatory genes [66,71]. Finally, our research group has recently demonstrated that it also prevents the alteration of subpopulations of neurogenic cells after KA insults [72].

### 4.2. The Metabolic-Cognitive Syndrome

Over the years, multiple studies have demonstrated a relationship between the appearance of cognitive deficits and phenomena like insulin resistance. This phenomenon causes impairments in glucose uptake both in central and peripheral regions. This alteration, which has a multifactorial origin, results in increased hepatic glucose production, favoring states of hyperglycemia and/or compensatory hyperinsulinemia in the periphery. Many pathologies like diabetes, cardiovascular disease, fatty liver disease, impaired lung function, mild cognitive impairment and Alzheimer’s disease have been linked with this pathologic condition [73,74,75,76,77].

Dr. Siegfried Hoyer was the first to mention this relationship. On his studies on the neuropathology of AD, he observed that a desensitization of the IRs might be a cause for the development of neurodegenerative hallmarks classically linked to dementia [78]. Later, the Rotterdam study revealed an increased risk of dementia in diabetic patients, stablishing a clear relationship between hyperglycemia and hyperinsulinemia states and the development of pathologies like AD [79]. Additionally, the analysis of human samples also yielded clear data demonstrating that AD patients show defective insulin signaling and response to this hormone just like altered levels and/or altered activation of components of the insulin signaling pathway [80].

#### 4.2.1. The Metabolism of Insulin

**Figure 5:**
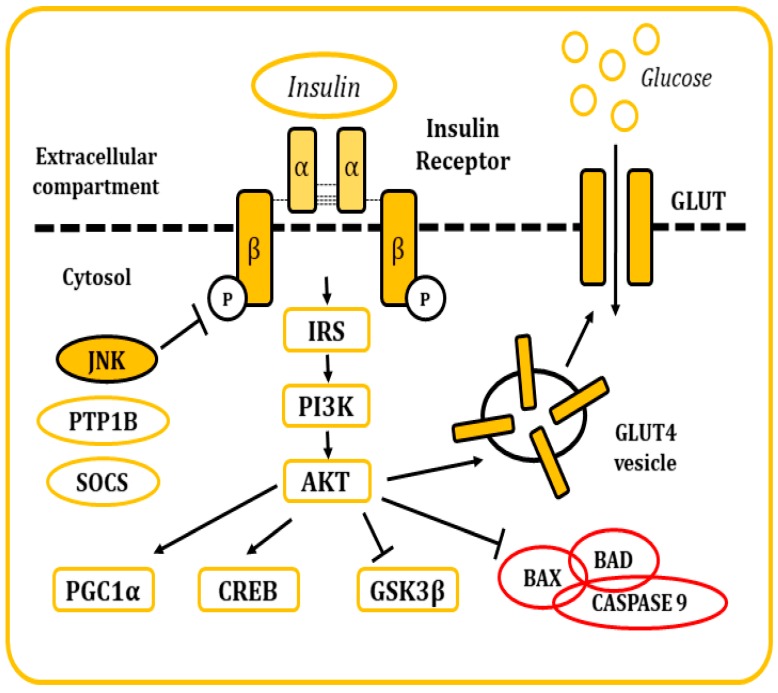
Representation of the cellular response after insulin stimulation. Insulin binds to the IR and causes its activation. The posterior signaling cascade stimulates the uptake of glucose through the GLUT transporters. This signaling pathway is also responsible for the regulation of multiple cellular functions and response mechanisms.

Insulin is a pancreatic polypeptide hormone produced by β cells in response to nutritional stimuli. It is responsible for the control of glucose concentrations in blood by stimulating their uptake into cells through its interaction with the IR [81,82]. All tissues depend on this mechanism for the maintenance of their metabolism, specially muscle, adipose tissue, liver, and the brain. It also plays a role in the control of development, cell growth, and division among other physiological functions [81,82,83]. The IR is a tetrameric tyrosine kinase protein made up of two extracellular α subunits and two transmembrane β subunits joined by disulphide bonds. On their resting state, α subunits behave as inhibitors of the tyrosine kinase activity of the β subunits. When insulin binds to the IR, this inhibition is relieved by the induction of conformational changes, allowing autophosphorylation of the IR in tyrosine residues [81,82,83,84]. Thereon, insulin receptor substrate (IRS) molecules are recruited and, when activated through tyrosine phosphorylation, they are responsible for signal transduction within the cell of a whole myriad of signals that will regulate cellular physiology [81,82,83,84,85,86,87,88] (Figure 5).

There are many regulatory mechanisms associated with the signaling of the IR that are involved in the maintenance of homeostasis. Yet, these mechanisms can also become dysregulated and be responsible for the development of pathological insulin resistance. In situations where insulin concentrations increase (hyperinsulinemia), the receptor becomes downregulated, internalized into vesicles, and, in some occasions, sent to lysosomal degradation [83]. IR signaling can also be inhibited by inflammatory cytokines, which in turn will activate stimuli response elements like IKB kinase β or the JNKs, mainly JNK1. These kinases have been described to phosphorylate the IRS molecules in serine residues, leading to the development of a decrease in insulin sensitivity and metabolic dysfunction [82,89,90]. Other elements responsible for the inhibitory control of insulin signaling include molecules like the protein tyrosine phosphatase 1B (PTP1B), responsible for the dephosphorylation of both IR and IRS proteins. Also, the suppressor of cytokine signaling (SOCS) proteins reduce IR signaling by either occupying the phoshotyrosine activity site on the IR or by recruiting ubiquitin ligases that will induce proteasomal degradation of IRS [91,92]. In the end, the dysregulation of these mechanisms is frequently associated with other conditions like obesity (visceral adiposity), systemic hypertension, hypercoagulation, and atherogenic dyslipidemia.

The occurrence of some or all of these alterations is referred to as metabolic syndrome. Worldwide, this condition is responsible for substantial morbidity and premature mortality [75]. Specifically, overweight and obesity are major risk factors for the development of metabolic syndrome, acting, in many cases, as the source or the adjuvant of the rest of the mentioned alterations [93,94].

#### 4.2.2. The Metabolism of Insulin in the Brain

**Figure 6:**
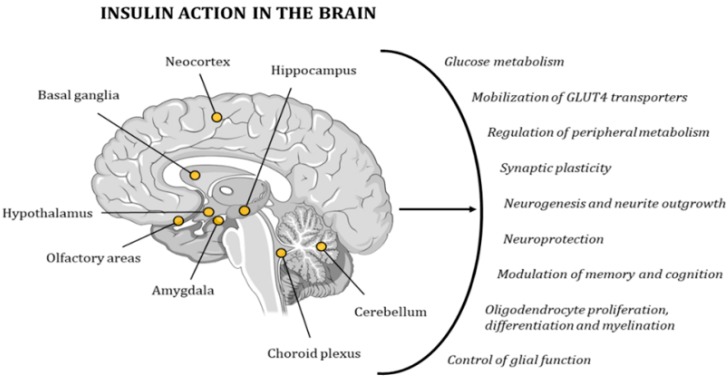
Insulin has a very strong effect on the functionality of the human brain, contributing to the maintenance of proper cognitive activity. The points in the image indicate the areas with the highest concentration in IRs.

Due to its wide distribution of IRs, the brain is highly sensitive to insulin. The highest concentration of these receptors can be found in the hypothalamus, olfactory areas, limbic regions, neocortex, basal ganglia, cerebellum, choroid plexus, and hippocampus [95,96,97,98]. Insulin activity controls glucose metabolism and the mobilization of GLUT4 transporters in neurons and glial cells. In addition, it regulates multiple metabolic pathways in peripheral tissues through the regulation of hypothalamic activity. Furthermore, this hormone has neuroprotective effects and a role in memory and cognitive modulation, since it modifies synaptic plasticity, and regulates neurogenesis and neurite outgrowth. It also regulates oligodendrocyte proliferation, differentiation, and myelination, as well as glial function (Figure 6) [98,99]. Hence, several studies have demonstrated the existence of a relationship between cognitive deficits and insulin resistance [78,79]. In 2005, Dr. Suzanne M de la Monte coined the term type 3 diabetes (T3D) to define a state of brain insulin resistance plus insulin deficiency that, in some cases, can overlap with type 2 diabetes mellitus (T2DM) [100,101]. In the last decade, many other researchers have backed up this hypothesis with other new discoveries and ground-breaking research projects [80,96,102,103,104].

#### 4.2.3. Role of JNKs in Metabolic-Related Dementias

One of the most striking elements of this pathologic scenario is that there is a decrease in the number of IRs in the blood brain barrier. This reduces the transport of insulin into the brain and causes a decrease in insulin action [105]. This situation favors mitochondrial dysfunction and ROS overproduction, as well as an increased activation of the UPR as part of the stress response [9,29,43,76,77,80,106]. Furthermore, activation of astrocytes and microglia leads to chronic inflammation, causing tissue damage and degeneration through the release of cytokines like TNFα, IL-1β, and IL-6, which later interact with receptors, such as the toll-like receptor 4 (TLR4) [39,80,96].

This pathologic paradigm is usually aggravated in obesity, where the excessive accumulation of adipocytes in perivisceral areas and the waist promotes an increase in the production of pro-inflammatory cytokines and higher blood levels of other lipidic molecules, such as sphingolipids and ceramides, which have been associated with the development of insulin resistance [106]. These dysregulations, and the subsequent cellular stress, increase the activation of stress-response elements like the JNKs. These molecules, in turn, phosphorylate IRSs in serine residues, feeding a vicious circle with further impairment of insulin signaling, as well as triggering the release, activation, and overproduction of pro-apoptotic molecules [3,32,43,76]. Other consequences of this overactivation of JNKs include an alteration of the hypothalamus-pituitary-thyroid axis and activation of PTP1B and SOCS3 molecules [43]. In addition, obesity-induced JNK1 activation leads to ER stress and induction of the UPR pathway by a mechanism that requires the double-stranded RNA-dependent protein kinase (PKR) [107,108]. Likewise, obesity could activate JNK1 through saturated fatty acids that might act as ligands for TLR4

In any case, eventually, dysregulations in metabolic activity have adverse effects on brain function and structure, inducing brain atrophy through the loss of grey matter, reduced integrity of white fiber tracts, white matter hyperintensity, infarcts, and microbleeds. Alterations can be either global or region specific, with a particularly detrimental effect in the medial temporal lobe, which includes the hippocampus. Other alterations include a loss of neurons, axodendritic pruning, reduced synaptic plasticity, and integrity, as well as decreased neurogenesis and cell proliferation, and a reduction in the number of dendritic spines. As a consequence of these events, impairments in learning, memory, and other cognitive functions may emerge [96,108].

## 5. JNKs as Potential Therapeutic Targets

Available preclinical data has demonstrated that modulation of the activity of the JNKs can have both beneficial and detrimental effects on insulin sensitivity, depending on the JNK isoform modulated and the tissue where the modification is occurring. For instance, whole body absence of JNK1 in KO models increases sensitivity to insulin and reduces body weight, even when animals are chronically exposed to a high-fat diet (HFD) [9,17,109,110]. Furthermore, these animals show reduced oxidative damage and inflammatory responses, as well as lower anxiety levels and increased neurogenesis [71,72,111]. Importantly, when conditional KO for JNK1 were produced in neurons, these animals showed even higher insulin sensitivity, as well as protection against the damage associated with chronic HFD feeding [112,113]. In turn, knockout of JNK3 under no metabolic stress has been shown to have no detrimental effects. Notwithstanding, when animals were treated with an HFD, severe hyperphagia due to leptin dysregulation was observed, causing a severe obesity phenotype [114]. Finally, whole-body ablation of JNK2 effects in brain and peripheral tissues have been less studied, and there is controversy over its actual effect on the control of energy metabolism. Relevantly, though, there is data on its importance in the maintenance of proper ER activity [115].

Based on the above, and considering that JNKs seem to play important roles in the development of many pathologic situations, multiple pharmacological options have been proposed with the objective of modulating the JNK pathway (Figure 6) [9,116]. Hence, initial research led to the synthesis of the first generation of chemical anthrapyrazole ATP competitive inhibitors like SP600125 and CEP1347. These small molecules were both tested in in vitro and in vivo models, and showed successful results in preventing JNK-dependent apoptosis mechanisms, reducing cytokine production, and improving recovery after ischemia/reperfusion damage [3,11]. Other studies demonstrated neuroprotective effects in models of stroke and spinal cord injury in mice [117]. Controversially though, these compounds showed poor bioavailability in the brain and had to be administered via intraventricular injections or infusions to achieve effective doses [116]. Moreover, they showed significant cellular toxicity, as well as lack of specificity; indeed, some of them have shown interactions with over 70 different molecules [11]. In addition, these compounds inhibited the phosphorylation of all substrates of JNKs, causing a lack of therapeutic effectivity in some models, or even aggravation of the pathology in others. For example, a treatment with SP600125 in mice suffering from transient focal cerebral ischemia 7 days before increased the infarct volume and inhibited vascular remodeling [116,117,118,119]. Hence, the potential clinical application of SP600125 has been impeded by its low target selectivity and its low aqueous solubility, whereas CEP1347 studies were discontinued due to their lack of efficacy or therapeutic capacity in patients for the treatment of Parkinson’s disease (ClinicalTrials.gov Identifier: NCT00040404, NCT00404170).

The second generation of ATP-competitive inhibitors like CC-401, AS601245, and Compound A showed lower toxicity but remained highly unspecific, rendering low therapeutic value [3,120]. In turn, a second wave of ATP non-competitive inhibitors’ synthesis focused on the function and structure of scaffold proteins like JIP. Specifically, these compounds were designed to target the substrate-docking site. In this respect, the D-JNKI-1 inhibitor peptide was demonstrated to be effective in a variety of pathological situations since it prevented downstream phosphorylation of JNK, and thus avoided the activation of pro-apoptotic transcription factors, such as c-JUN and c-FOS. In addition, it reduced the production of amyloid β and exerted neuroprotective effects in a model of myocardial ischemic injury [121,122]. The results of these studies seemed to indicate high target specificity [116]. Likewise, a high JNK3 inhibitory capacity of the D-JNKI-1 peptide was observed in mitochondria [123]. This molecule has shown increased specificity and favorable results in a wide spectrum of pathologies related to hearing loss caused by wide cochlear damage and amyminoglycoside ototoxicity mainly due to otoprotective effects [121,122,123,124] (Table 1).

Moreover, two new compounds, namely SR3306 and SR11935, which are selective, water-soluble, and able to cross the blood brain barrier, have been developed [125]. At a preclinical level, both compounds can suppress feeding and reduce body weight, just like improved situations of leptin resistance under high-fat diet feeding conditions. Furthermore, Jang and colleagues reported that two JNK selective inhibitors, SU3327 and BI-78D3, which potently blocked JNK, protect against hepatotoxicity mediated by CCl4 (50 mg/kg, IP) [126]. Also, Plotnikov and colleagues demonstrated that a specific JNK inhibitor, IQ-1S (11H-indeno [1–b]-quinoxalin-11-one oxime sodium salt), which has a higher affinity for JNK3 compared with its affinity to JNK1/JNK2, showed a neuroprotective effect in models of ischemia in rat [127].

Yet, in spite of these promising results, one of the main issues is that these inhibitors are not tissue specific. In addition, they inhibit all JNK isoforms, which can be the grounds for the appearance of a lack of therapeutic effect or even a worsening of the pathological situation. The use of experimental murine models has shown that JNK isoforms have, in some occasions, overlapping and redundant functions, but also that, in many others, each isoform has a specific role that varies in a cell-type and signal-dependent manner. For example, JNK1 and JNK2 have shown opposite effects on the turnover and activity of c-JUN [120]. Furthermore, a preclinical model of joint damage in mice demonstrated that JNK1 deficiency led to a reduction of inflammatory cell infiltration while a lack of JNK2 showed no benefit [128,129,130]. In another study, Kamenecka and colleagues aimed to synthesize a selective JNK3 inhibitor with neuroprotective effects, but the compounds inhibited cytochrome P450, an essential component of cellular metabolism, which made them non-viable due to the high risk of negative side effects [131]. Indeed, the design and synthesis of isoform-specific inhibitors is highly complicated. Itt is worth mentioning that within the ATP binding pocket, JNK1 and 2 can only be differentiated in two positions in the amino acidic sequence, whereas only one point of variation exists between JNK1 and 3 and between JNK2 and 3 [17].

Nevertheless, some natural molecules have been described as isoform-specific inhibitors. Hence, compounds like latifolians A and B, which are benzylisoquinolones extracted from the *Gnetum latifolium* plant (found in Papua New Guinea), behave as specific JNK3 inhibitors [132]. Licochalcone A (Lic-A) is a chalcone phenolic component found in the roots of licorice (*Glycyrrhiza inflata*), which has been described as a specific inhibitor of the JNK1 isoform [133]. Like many traditional herbal medicines and foods, it also exhibits anti-inflammatory and antioxidative effects. Mechanistically, Lic-A competes with JIP for binding of JNK1 and causes distortions in the conformation of the ATP binding cleft, thus reducing its activity. A dosage of 20 mg/kg has proven to have beneficial anticancer effects and to be well tolerated by mice [133]. However, an excessive daily dosage of 50 g can lead to a significant rise in blood pressure and thus to negative effects [133,134].

## 6. Concluding Remarks

Although the scientific community has unveiled many aspects of JNK-dependent mechanisms and their role in pathological situations, a complete understanding of these major signaling cascades is yet to come [135,136,137,138]. The present review aimed to describe the role of JNKs in the pathophysiology of TLE and metabolic-cognitive affectations. We also discussed how targeting JNKs could be of beneficial interest. Thus, JNK1 inhibition has proven to exert significant beneficial effects, such as neuroprotection, neuroinflammatory modulation, and prevention of type 2 diabetes and obesity [133,134]. Regarding the last one, obesity plays an important role in JNK1 activation and inhibition of insulin receptors, hence increasing the risk of insulin resistance and contributing to cognitive impairment. Although early studies have provided an important insight into both the peripheral metabolic role and brain regulatory role of JNK, many issues remain to be solved. Firstly, the specific mechanisms by which obesity alters the JNK pathway and increases the risk of cognitive loss in the hippocampus remains to be elucidated [135,136,137,138,139]. Secondly, although hypothalamic signaling pathways involving JNK–dependent regulation of peripheral metabolism have been determined, the way these pathways influence other brain areas, such as the hippocampus, under stress conditions or vice versa is still not fully understood [140,141].

The use of animal models and clinical trials will help to define the function of JNK in epilepsy, neurodegenerative diseases, and obesity. So far, it can be stated that the neuroinflammatory response triggered by JNK activation could be involved in a loss of synapses, neuronal cell death, and cognitive impairment. However, JNK is also involved in key cellular physiologic aspects [142,143,144,145,146,147]. Hence, in the future, it will be necessary to examine the molecular mechanisms underlying the JNK function, both under physiologic and pathological conditions, paying special attention to crosstalk among these pathways. We hypothesize that a better characterization of JNK activity in epilepsy, neurodegeneration, and obesity will allow to the development of specific drugs with clinical relevance.

## Figures and Tables

**Table 1:** Current clinical trials assessing D-JNKI-1 for the treatment of hearing and ocular diseases.

Drug	Phase	CT Number	Disease
D-JNKI-1	III	NCT02809118	Idiopathic sudden sensorineural hearing loss
D-JNKI-1	III	NCT02235272	Post-cataract Surgery Intraocular Inflammation
D-JNKI-1	I	NCT01570205	Safety, Tolerability and PK of a Single iv Infusion of 10, 40, and 80 µg/kg XG-102 Administered to Healthy Volunteers
D-JNKI-1	III	NCT02561091	Acute Inner Ear Hearing Loss
D-JNKI-1	III	NCT02508337	Reduction of Post-cataract Surgery Intraocular Inflammation and Pain
D-JNKI-1	II	NCT00802425	Acute Sensorineural Hearing Loss
